# Evaluation of magnetic resonance imaging abnormalities in juvenile onset neuropsychiatric systemic lupus erythematosus

**DOI:** 10.1007/s10067-016-3376-9

**Published:** 2016-08-15

**Authors:** M Al-Obaidi, D. Saunders, S. Brown, L. Ramsden, N. Martin, E. Moraitis, C. A. Pilkington, P. A. Brogan, D. Eleftheriou

**Affiliations:** 1 Paediatric Rheumatology, Great Ormond Street Hospital for Children NHS Foundation Trust, London, UK; 2 Radiology Department, Great Ormond Street Hospital for Children NHS Foundation Trust, London, UK; 3 Department of Paediatric Rheumatology, Great Ormond Street Hospital for Children NHS Foundation Trust and UCL Institute of Child Health, London, WC1E1HN UK

**Keywords:** Juvenile SLE, Magnetic resonance imaging, Neuropsychiatric SLE

## Abstract

The aim of this study was to describe the abnormalities identified with conventional MRI in children with neuropsychiatric systemic lupus erythematosus (NPSLE). This was single-centre (Great Ormond Street Hospital, London) retrospective case series of patients with juvenile NPSLE seen in 2003–2013. Brain MR images of the first episode of active NPSLE were reviewed. All patients fulfilled the 1999 ACR case definitions for NPSLE syndromes. Presenting neuropsychiatric manifestations, immunological findings and treatment are reported. Results are expressed as median and ranges or percentages. Fisher’s exact test was used to identify clinical predictors of abnormal MRI. A total of 27 patients (22 females), median age 11 years (4–15), were identified. Presenting clinical symptoms included the following: headaches (85.1 %), mood disorder/depression (62.9 %), seizures (22.2 %), acute psychosis (18.5 %), cognitive dysfunction (14.8 %), movement disorder (14.8 %), acute confusional state (14.8 %), aseptic meningitis (7.4 %), demyelinating syndrome (3.7 %), myelopathy (3.7 %), dysautonomia (3.7 %) and cranial neuropathy (3.7 %). The principal MR findings were as follows: (1) absence of MRI abnormalities despite signs and symptoms of active NPSLE (59 %); (2) basilar artery territory infarction (3 %); (3) focal white matter hyperintensities on T2-weighted imaging (33 %); (4) cortical grey matter lesions (3 %); and (5) brain atrophy (18.5 %). The presence of an anxiety disorder strongly associated with abnormal MRI findings (*p* = 0.008). In over half the children with NPSLE, no conventional MRI abnormalities were observed; white matter hyperintensities were the most commonly described abnormalities. Improved MR techniques coupled with other alternative diagnostic imaging modalities may improve the detection rate of brain involvement in juvenile NPSLE.

**Electronic supplementary material:**

The online version of this article (doi:10.1007/s10067-016-3376-9) contains supplementary material, which is available to authorized users.

## Introduction

Systemic lupus erythematosus (SLE) is a common autoimmune disease that presents before the age of 16 years old in approximately 10–20 % of cases, and in that context is referred to as juvenile onset SLE (jSLE) [1]. Neuropsychiatric SLE (NPSLE) presents with a frequency between 14 and 25 % in children; neuropsychiatric symptoms often develop within the first year of SLE diagnosis and are related to high mortality [2–4]. In 1999, the American College of Rheumatology (ACR) established nomenclature and detailed case definitions for 14 NPSLE syndromes, which provide a clear description of the multiple clinical faces of the disorder and standardised the classification of adults with NPSLE (supplemental Table 1) [5]. Although paediatric input was minimal, these definitions are commonly applied to paediatric patients in daily practice and utilised in clinical research [3, 5]. However, correct attribution of neuropsychiatric events to NPSLE or to an alternative aetiology is a challenge, given the absence of a diagnostic gold standard for NPSLE.

As magnetic resonance imaging (MRI) is widely available, it is usually the imaging technique of choice for the work-up of NPSLE [3, 6]. A wide variety of MRI findings in adults with NPSLE have been previously described and are suggestive of different NPSLE pathogenetic mechanisms [6, 7]: (1) focal hyperintensities in white matter or both white and grey matter, suggestive of vasculitis, or multifactorial autoimmune-mediated mechanisms of vascular occlusion or narrowing (vasculopathy with ischaemia); (2) more widespread, confluent hyperintensities in the white matter, suggestive of chronic hypoperfusion due to the same mechanisms; (3) diffuse cortical grey matter lesions, compatible with an autoantibody-mediated immune response to neuronal or other CNS components, or postseizure changes; and (4) no conventional MRI abnormalities at all, despite signs and symptoms of active disease [6, 7]. However, the role of MRI in the evaluation of children and adolescents with suspected juvenile NPSLE remains largely unknown.

The aim of this study was therefore to (i) describe the abnormalities seen on conventional MRI in a large group of children with jSLE, during episodes of active NPSLE manifestations; (ii) identify clinical predictors of abnormalities seen on conventional MRI.

## Patients and methods

### Patients

We retrospectively screened the case notes of a total of 323 patients with jSLE seen at the paediatric rheumatology department at Great Ormond Street Hospital for Children NHS Foundation Trust between April 2003 and October 2013, for patients fulfilling the 1999 ACR case definition for NPSLE syndromes [8], and in whom at least one MR imaging study had been performed. All patients met at least 4 of the 11 ACR revised criteria for the classification of SLE [8]. Patients with neuropsychiatric symptoms that could easily be attributed to causes other than jSLE were excluded such as patients with previous existing epilepsy, infection, traumatic brain injury, electrolyte abnormalities, or medication side effects. Ethical approval was given by the Institute of Child Health/Great Ormond Street Research Ethics Committee for a retrospective case notes review; since this was a retrospective case note review using de-identified data, written consent from patients was not required.

The demographic, clinical, and laboratory characteristics recorded at diagnosis of NPSLE were as follows: sex, age, ethnicity (established via the information provided by patients/parents to register with the hospital), previous organ involvement, manifestations of NPSLE, erythrocyte sedimentation rate (ESR, in the first hour; normal <10 mm/h) and serum C-reactive protein (CRP; normal range < 10 mg/L), presence of anti-dsDNA antibodies (normal range 0–20 iu/ml), antiphospholipid antibodies and lupus anticoagulant, complement C3 and C4 levels. Therapies used for NPSLE manifestations were also recorded.

### MRI acquisition

**Fig. 1:**
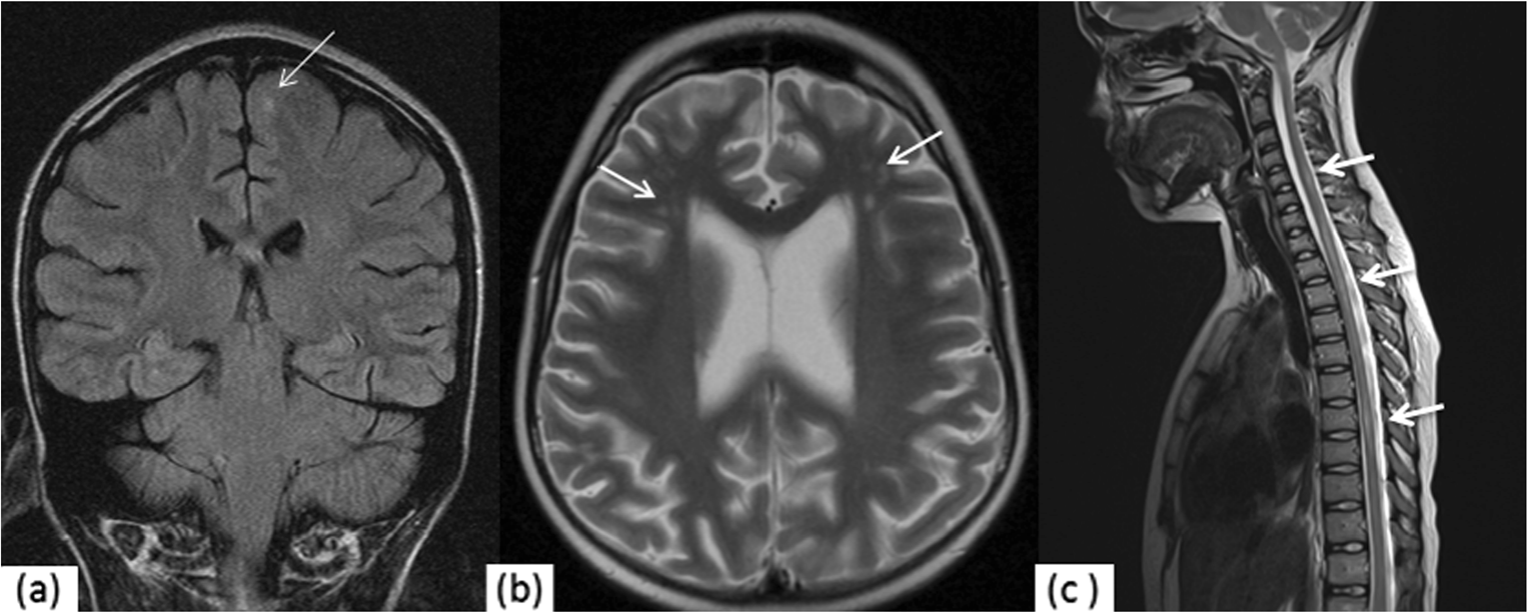
**a** Representative example of a grey matter signal hyperintensity (*arrow*) seen on the coronal FLAIR image (*long arrow*). **b** Clusters of T2 hyperintensities (*arrows*) in the frontal white matter seen on T2-weighted images. Note the background of sulcal and ventricular enlargement possibly resulting from cerebral atrophy. **c** Sagittal T2 hyperintensity of the cord (*arrows*) that resolved and remitted over a number of years. *FLAIR* fluid-attenuated inversion recovery

MRI and MRA were performed on a 1.5-T MR scanner using a standard imaging protocol, including T2-weighted turbo spin-echo imaging in the axial plane, fluid-attenuated inversion recovery (FLAIR) sequence in the coronal plane, T1-weighted spin-echo imaging in the sagittal plane and three-dimensional short-echo time-of-flight MRA of the circle of Willis in some patients. When available, additional sequences such as T1-weighted images following intravenous gadolinium (Gd) administration, diffusion-weighted images and apparent diffusion coefficient maps were also reviewed. An experienced neuroradiologist (SB) blinded to the clinical status of the patients examined all images for the presence of any abnormality. Lesions were categorised as hyperintensities (i.e. showing high signal intensity on T2-weighted images, proton density-weighted images and/or FLAIR images), parenchymal defects or areas of focal atrophy (Fig. 1 shows some of the abnormalities). Parenchymal defects were areas of T2 hyperintensity usually associated with T1 hypointensity. Atrophy was defined as areas of brain parenchyma characterised by volume loss between two scans or as enlarged CSF spaces for a patient of their given age on a single scan. For each lesion, the behaviour following Gd administration were documented. Depending on their location, lesions were characterised as cortical grey matter (GM), supratentorial white matter (WM), basal ganglia, brainstem, spinal cord or cerebellar WM and GM lesions.

### Statistical analysis

Descriptive statistics were used to describe the study cohort: continuous variables were summarised as median and range; categorical variables were presented as percentages. Parameters between groups were compared using the Fisher’s exact test. A two-sided *p* value <0.05 was considered statistically significant. Statistical analysis was done with IBM SPSS statistics version 21.

## Results

### Demographics, jSLE and NPSLE features

A total of 27 patients (22 females) of median age of 11 years (range 4–15) with NPSLE were included in the study. Eleven out of 27 (40.7 %) were Afro-Caribbean; 4/27 (14.8 %) were Pakistani; 7/27 (25.9 %) were Caucasian; and the remaining 4/27 (14.8 %) were of mixed ethnic background.

The median age of jSLE disease onset was 11 years old (range 4–15), and the median time from diagnosis to the development of NPSLE symptoms was 16 months (range 0–70). The jSLE disease features from diagnosis to the time patients developed suspected NPSLE are summarised in Table 1.

**Table 1:** Summary of juvenile onset systemic lupus erythematosus (jSLE) disease features from diagnosis to the time patients developed neuropsychiatric syndromes. Definitions are based on the revised American College of Rheumatology (ACR) criteria for classification of SLE [8]

Clinical manifestations	*N* of 27 patients (% of total)
Malar rash	21 (77 %)
Discoid lupus	10 (37 %)
Photosensitivity	11 (40 %)
Oral or nasal ulceration	10 (37 %)
Arthritis	17 (62 %)
Serositis	14 (51 %)
Nephritis	18 (66 %)
Neurological disease	27 (100 %)
Haematological disorder	19 (70 %)

Persistent severe headaches were the most frequent neuropsychiatric manifestation, occurring in 23/27 (85.1 %) patients, followed by mood disorder/depression in 17/27 (62.9 %); anxiety disorder in 7/27 (25.9 %); seizures in 6/27 (22.2 %); acute psychosis in 5/27 (18.5 %); cognitive dysfunction in 4/27 (14.8 %); movement disorder in 4/27 (14.8 %); acute confusional state in 4/27 (14.8 %); aseptic meningitis in 2/27 (7.4 %); demyelinating syndrome 1/27 (3.7 %); myelopathy 1/27 (3.7 %); dysautonomia 1/27 (3.7 %); and cranial neuropathy in another patient (3.7 %).

### Laboratory and other investigations

Antinuclear antibodies were positive in all 27 patients, and anti-dsDNA antibodies were present in 20/27 (74 %) of patients, at a median level of 14 iu/ml (range 0.5–400). Low C3 levels were documented in 14/27 (51.8 %) patients and low C4 in 15/27 (55.5 %). ENA antibodies were detected in 14/27 patients (51.8 %): anti-Ro antibodies in 8 patients; anti-La in 2 patients (both positive for anti-Ro as well); 6 patients were positive for anti-RNP, and 2 of these were also positive for anti-SM antibodies. IgG anticardiolipin antibodies (ACL) were positive in 10 out of 27 (37 %) patients at the time of acute NPSLE; lupus anticoagulant was positive in 4/27 (14 %; all positive for IgG ACL as well); and anti-β2 glycoprotein antibodies were positive in 1/11 patients tested (positive for lupus anticoagulant too). Median ESR was 24 mm/h (range 10–75); median CRP 25 mg/L (5–45).

CSF examination was performed in two patients: this demonstrated pleocytosis in both cases (mainly lymphocytes), with CSF normal protein and glucose levels, and negative CSF culture in both cases.

Electroencephalography (EEG) was performed in 11/27 (40 %) patients; a total of 5/11 of these cases had presented with seizures. EEG was abnormal in 2/11 patients tested, with EEG showing generalised spike waves.

### MRI abnormalities and NPSLE presentation

A total of 27 imaging studies were reviewed; gadolinium contrast-enhanced images were available in 23/27cases. Diffusion-weighted images were available for 21/27 MRI scans.

Sixteen of 27 (59.3 %) patients spanning eight different ACR NPSLE manifestation categories had no MRI abnormality demonstrated in both precontrast and postcontrast images (available in all 16 cases). Their NPSLE syndromes were seizures (*n* = 2), cerebrovascular disease (*n* = 1), headaches (*n* = 14), movement disorder (*n* = 1), cognitive disorders (*n* = 2), psychiatric disorders (*n* = 11), and cranial neuropathy (*n* = 1). Three of these 16 patients also had cerebral MRA; this was normal in all cases. Ten of 16 patients also had diffusion-weighted imaging scans; normal diffusion was noted in all.

**Fig. 2:**
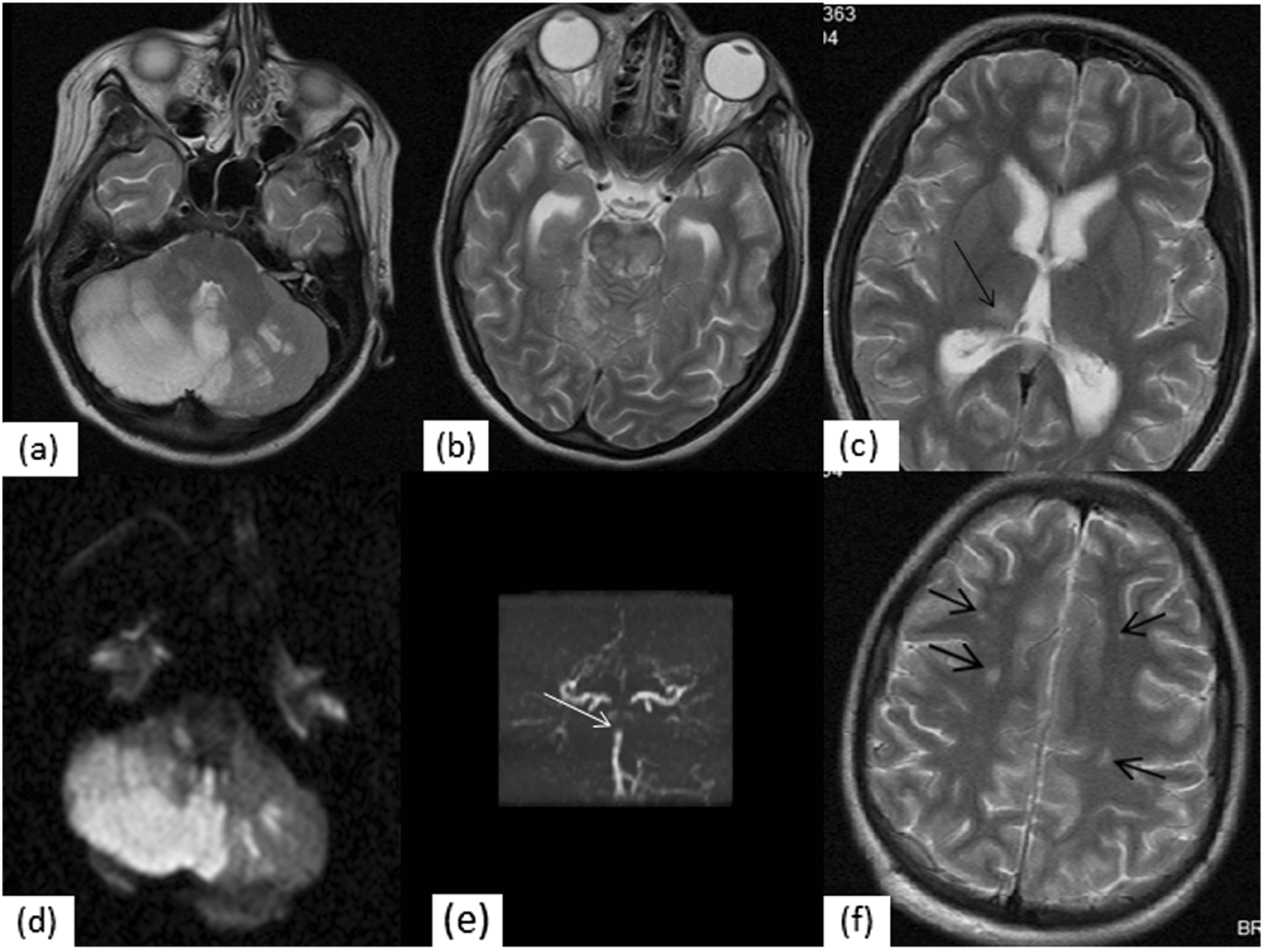
Infarction in a 14 year old with NPSLE of the **a** cerebellum, **b** brainstem, and **c** right posterior thalamus (*arrow*) diagnosed on the T2-weighted MR images. Cerebellar swelling compressing the fourth ventricle resulted in mild hydrocephalus. **d** The diffusion-weighted imaging revealed a high signal lesion in keeping with an acute infarct. **e** A signal void of the distal basilar artery (*white arrow*) on the MRA of the posterior circulation revealed a large vessel occlusion. **f** Multiple borderzone infarcts are seen within the deep white matter of the frontal lobes, some of which were acute. The anterior circulation was normal (not shown)

MRI scans were abnormal in the remaining 11 patients presenting with seizures (*n* = 4), aseptic meningitis (*n* = 2), cerebrovascular disease (*n* = 2), demyelination (*n* = 1), headaches (*n* = 9), movement disorder (*n* = 3), transverse myelopathy (*n* = 1), cognitive disorders (*n* = 3), psychiatric disorders (*n* = 8), autonomic disorder (*n* = 1), peripheral neuropathy (*n* = 1), and myasthenia-like syndrome (*n* = 1). No patients in our cohort had sensorineural hearing loss. The most frequent radiographic finding in our 11 patients with abnormal scans was hyperintensity on T2 in 9/11 (81.8 %). One or more white matter hyperintensities were observed in 8/11 (72.2 %) of NPSLE patients with other MRI abnormalities occurring in various numbers and sizes. One child had an acute basilar artery occlusion resulting in infarction of the cerebellum, brainstem, posterior thalamus on the right and mild hydrocephalus with restricted diffusion (Fig. 2). Of the remaining, white matter hyperintensity lesions occurred in the supratentorial white matter in 7/8 patients (88.9 %), in the brainstem in one patient (11.1 %). Multiple white matter lesions these were distributed asymmetrically. White matter lesions were present in patients with all types of ACR-defined NPSLE syndromes in this study (Table 2).

**Table 2:** Magnetic resonance imaging (MRI) findings corresponding to ACR-defined neuropsychiatric syndromes associated with systemic lupus erythematosus (NPSLE). Each patient may appear under more than one category of NPSLE syndrome

			Hyperintensity					Parenchymal defect			Atrophy	
NPSLE syndrome	Patients	Abnormal MRI	Supratentorial white matter	Cortical				Supratentorial white matter	Basal ganglia	Cerebellum	Supratentorial white matter	Cortical grey matter
				Brainstem	Grey matter	Basal ganglia	Cerebellum					
Seizure disorder	6	4	3	0	1	0	1	0	0	1	3	1
Aseptic meningitis	2	2	1	0	1	0	0	0	0	0	1	1
Cerebrovascular disease	3	2	2	0	0	0	1	0	0	1	2	0
Demyelinating syndrome	1	1	0	1	0	0	0	0	0	0	0	0
Headache	23	9	7	1	1	0	1	0	0	1	3	1
Movement disorder	4	3	2	1	0	0	1	0	0	1	1	0
Transverse myelopathy	1	1	0	1	0	0	0	0	0	0	0	0
Cognitive disorders												
Acute confusional state	4	3	2	0	1	0	1	0	0	1	2	1
Cognitive dysfunction	4	3	3	0	1	0	1	0	0	1		
Psychiatric disorders												
Anxiety disorder	7	6	5	0	1	0	1	0	0	1	3	1
Mood disorder/depression	17	8	7	0	1	0	1	0	0	1	3	1
Psychosis	5	4	3	0	1	0	1	0	0	1	2	1
Autonomic disorder	1	1	0	1	0	0	0	0	0	0	0	0
Peripheral neuropathy	1	1	0	1	0	0	0	0	0	0	0	0
Cranial neuropathy	1	0	0	0	0	0	0	0	0	0	0	0
Myasthenia-like syndrome	1	1	0	1	0	0	0	0	0	0	0	0
Sensorineural hearing loss	0	0	0	0	0	0	0	0	0	0	0	0

Grey matter hyperintensities were observed in 1/11 patients affecting the cortex (Fig. 1). This patient also showed multiple, similar-appearing, focal white matter lesions. There was also restricted diffusion in the scan.

One or more parenchymal defects were detected in a single patient; lesions were located in the cerebellum and were in the presence of other white matter or grey matter lesions.

One child had T2 hyperintensities in the cervicothoracic cord that came and went over a number of years and was diagnosed as having a transverse myelitis (Fig. 1c).

Another child had an extensive acute infarction of the midbrain and both cerebellar hemispheres with ischaemic change in the deep white matter watershed regions of both cerebral hemispheres, body of the corpus callosum and left parietal cortex (Fig. 2). MRA revealed occlusion of the distal basilar artery.

Cerebral atrophy was diagnosed in five patients; this was located in the supratentorial white matter in 4/5 patients and in the cortical grey matter in one patient.

Regarding post-Gd administration images, there was no contrast enhancement in 19/24 scans including no enhancement of the infarcted area for the patient with the basilar artery infarction. There was patchy enhancement over the spinal lesions for the patient with transverse myelitis; parotid gland enhancement for one patient with no intracerebral lesion enhancement; and enhancement over a small incidental developmental venous anomaly for another patient.

### Clinical predictors of abnormal brain MRI

There was a significant association between the presence of an anxiety disorder and abnormal brain MRI scans (*p* = 0.008). There was no specific association between other NPSLE manifestations, disease duration, presence of ACL and presence of cerebral abnormalities on MRI (Table 3).

**Table 3:** Predictors of abnormal brain magnetic resonance imaging (MRI) in patients with neuropsychiatric syndromes associated with systemic lupus erythematosus (NPSLE). Groups were compared using Fisher’s exact test; *p* < 0.05 was considered significant. *ACL* anticardiolipin antibodies

Presence of NPSLE syndrome	Normal MRI	Abnormal MRI	*p* value
Seizure disorder			
Yes	2	4	0.187
No	14	7	
Aseptic meningitis			
Yes	0	2	0.156
No	16	9	
Cerebrovascular disease			
Yes	1	2	0.548
No	15	9	
Demyelinating syndrome			
Yes	0	1	0.4
No	16	10	
Headache			
Yes	14	9	1
No	2	2	
Movement disorder			
Yes	1	3	0.27
No	15	8	
Transverse myelopathy			
Yes	0	1	0.4
No	16	10	
Cognitive disorders			
Acute confusional state			
Yes	1	3	0.27
No	15	8	
Cognitive dysfunction			
Yes	1	3	0.27
No	15	8	
Psychiatric disorders			
Anxiety disorder			
Yes	1	6	0.008
No	15	5	
Mood disorder / depression			
Yes	9	8	0.44
No	7	3	
Psychosis			
Yes	1	4	0.125
No	15	7	
Autonomic disorder			
Yes	0	1	0.4
No	16	10	
Peripheral neuropathy			
Yes	0	1	0.4
No	16	10	
Cranial neuropathy			
Yes	1	0	1
No	15	11	
Myasthenia-like syndrome			
Yes	0	1	1
No	16	10	
Disease duration			
<12 months	9	6	1
>12 months	7	5	
Presence of ACL			
Yes	7	3	0.44
No	9	8	

### Treatment

The treatment received by the 27 patients for the suspected NPSLE included: intravenous corticosteroids (30 mg/kg/day for 3 days followed by 2 mg/kg of oral prednisolone tapered over 3–6 months) in all patients; intravenous cyclophosphamide (500 mg–750 mg/m^2^ monthly over 6 months) in 14/27 (51.8 %) patients; rituximab (750 mg/m^2^ two doses repeated in 2 weeks) in 11/27 (40.7 %) patients; and plasma exchange (two volume exchanges per day over 5 cycles) in 3/27 (11.1 %) patients. Four of 27 (14.8 %) patients received aspirin as an antiplatelet agent; 6/27 patients (22.2 %) received anticoagulation therapy (warfarin or low-molecular-weight heparin); all these patients were positive for IgG ACL. A total of 4 of the 6 patients with seizures received antiepileptic medication that was discontinued after 12–18 months.

## Discussion

In this observational study, we describe the cerebral abnormalities detected using MRI during active NPSLE episodes in jSLE. Surprisingly, brain MRI was normal in 59 % of our patients, an observation we suggest is clinically important since it emphasises that a normal brain MRI does not exclude potentially reversible NPSLE. Presentation with an anxiety disorder strongly associated with the presence of an abnormal MRI, despite patients not presenting with any focal neurological manifestations. This finding is also of considerable clinical relevance since physicians may dismiss anxiety in this context as being a feature of chronic illness and its treatment, rather than due to potentially reversible organic brain disease per se.

The 59 % of patients with normal MRI scans exhibited a wide variety of NPSLE symptoms. This is in line with previous studies in adults with NPSLE that demonstrated up to 42 % of patients with active NPSLE had a normal MRI [7]. This observation highlights the limitations of conventional MR scanning used in routine clinical care to detect brain abnormalities, despite strong clinical suspicion of NPSLE. Better neuroimaging techniques for NPSLE that are capable of identifying the underlying pathological mechanism (inflammatory and thrombotic) are needed to facilitate the diagnosis of NPSLE, particularly since treatment delay can be associated with high morbidity and mortality. Novel functional and quantitative MRI techniques such as magnetic resonance spectroscopy (MRS), magnetisation transfer imaging (MTI), diffusion-weighted imaging (DWI), and perfusion-weighted imaging (PWI), as well as nuclear imaging techniques, such as single-photon emission computed tomography (SPECT) may have a role in detecting changes in juvenile NPSLE, and could facilitate earlier diagnosis [9–12]. Quantitative MRI techniques are also now beginning to reveal abnormalities in NPSLE patients with normal conventional MRI scans [10]. The clinical relevance of these approaches therefore requires further validation in jSLE.

Our observations in jSLE concur with those of previous studies in adults showing that white matter hyperintensities were the most commonly observed lesion in patients with an abnormal MRI scan [6, 7]. Notably, white matter lesions have also been shown to be present in some patients without NPSLE, suggesting that these lesions are common and their specificity remains to be established [6]. In addition, and whilst the exact pathophysiology of these NPSLE-related focal white matter hyperintensities remains unclear, previous reports demonstrated a strong correlation between the presence of these lesions and scores of NPSLE disease activity, emphasising their clinical relevance and suggesting an aetiological link [13, 14].

We did not have many patients with infarcts due to thromboembolic occlusion of any medium or large arteries (*n* = 1), in contrast to studies in adults with NPSLE [6]. Additional factors such as higher incidence of antiphospholipid syndrome in adults, and/or accelerated atherosclerosis may explain this observed difference.

Our study is limited by all the usual caveats around small, largely descriptive retrospective case studies, an unavoidable situation since NPSLE is so rare in the young. We used standardised protocols for conventional MRI sequences (such as T1-weighted [with and without contrast agents], T2-weighted, and FLAIR images), as utilised for routine care in our institution for at least 10 years. Further characterisation of the lesions with more recently introduced techniques such as diffusion-weighted imaging was performed for the majority of the patients but not for all cases and may have increased the MRI detection yield. We have also not tested for the presence of recently described antineuronal antibodies that have been demonstrated in some patients with jSLE [15], since these tests were not routinely available at our institution.

In summary, normal conventional MRI scans were observed in 59 % of our patients with clinically active NPSLE. Therefore, a normal conventional MRI study cannot reliably exclude a diagnosis of NPSLE. For the remaining 41 %, we observed several distinct radiographic patterns likely reflective of different NPSLE pathogenic mechanisms. Anxiety disorder strongly associated with the presence of an abnormal MRI. Improved neuroimaging techniques that combine morphological with functional imaging may in the future improve the detection rate of brain involvement in juvenile NPSLE.

## Electronic supplementary material

ESM 1 (DOCX 16 kb)
