# Supplementary material for: Evaluation of magnetic resonance imaging abnormalities in juvenile onset neuropsychiatric systemic lupus erythematosus
Source: Clin Rheumatol. 2016 Aug 15;35(10):2449–56. doi: 10.1007/s10067-016-3376-9 (PMC5031744; doi:10.1007/s10067-016-3376-9)
Supplement: Supplementary file 1 — (DOCX 16 kb) [file 10067_2016_3376_MOESM1_ESM.docx]

**Supplemental table online 1.** The American College of Rheumatology (ACR) definitions for Neuropsychiatric Syndromes Associated with Systemic lupus erythematosus (NPSLE) (5).

| Neuropsychiatric Syndromes Associated With Systemic lupus erythematosus (NPSLE) |
| --- |
| \| NPSLE ASSOCIATED WITH CENTRAL NERVOUS SYSTEM   - Aseptic Meningitis - Cerebrovascular disease   - Stroke   - Transient Ischaemic Attack   - Cerebral Venous Sinus Thrombosis - Cognitive Disorders   - Delirium (Acute confusional state)   - Dementia   - Mild Cognitive Impairment - Demyelinating syndromes - Headaches   - Tension Headaches   - Migraine Headaches - Movement disorders (Chorea) - Psychiatric Disorders   - Psychosis   - Mood Disorders   - Anxiety Disorder - Seizure Disorders - Transverse Myelopathy \| \| --- \| \| NPSLE ASSOCIATED WITH PERIPHERAL NERVOUS SYSTEM   - Autonomic Neuropathy - Myasthenia Gravis - Peripheral neuropathy - Sensorineural Hearing Loss   - Sudden Onset   - Progressive \| \| Cranial neuropathy \| |
